# Placental DNA Methylation Abnormalities in Prenatal Conotruncal Heart Defects

**DOI:** 10.3389/fgene.2022.878063

**Published:** 2022-05-13

**Authors:** Jingjing Liu, Yuduo Wu, Hairui Sun, Xiaowei Liu, Xiaoyan Gu, Ying Zhao, Ye Zhang, Jiancheng Han, Yihua He

**Affiliations:** ^1^ Echocardiography Medical Center, Beijing Anzhen Hospital, Capital Medical University, Beijing, China; ^2^ Maternal-Fetal Medicine Center in Fetal Heart Disease, Beijing Anzhen Hospital, Capital Medical University, Beijing, China

**Keywords:** placenta, methylation, biomarkers, epigenetics, conotruncal heart defects

## Abstract

**Objective:** This study aims to characterize the abnormal changes in placental DNA methylation associated with conotruncal heart defects (CTDs) and the level of methylation as epigenetic biomarkers for CTDs detection.

**Methods:** This was a prospective study involving 28 fetuses diagnosed with CTDs in the second trimester at Beijing Anzhen Hospital between September 2020 and June 2021. These cases were classified into four groups based on their subtypes. 12 normal fetuses were used as controls. Placental tissue was obtained after inducing labor in fetuses. To identify differential methylation sites (DMSs) and regions (DMRs) in cases vs. controls, an Infinium Human Methylation 850 k bead chip was used. Differential methylation was assessed by comparing the β-values for individual CpG loci. Based on the p-value (<0.05), the most discriminating CpG sites were identified. The area under the receiver-operating-characteristics curve (AUC) was used to determine the predictive accuracy of CpG loci with significant methylation changes for CTDs. The function of genes was assessed through KEGG enrichment analysis, Gene Ontology (GO) analysis, and KEGG pathway analysis.

**Results:** In comparison to the control group, the DNA methylation of the placental tissue is significantly different in fetuses with CTDs. We identified the most significantly different methylated loci and they demonstrated excellent individual predictive accuracy for CTDs detection with AUC >0.9 in cases compared with controls. *HOXD9, CNN1, NOTCH1*, and *ECE1* were identified as CTDs-detection candidate genes.

**Conclusion** Our study established the abnormal changes in placental methylation associated with CTDs and potential epigenetic biomarkers for CTDs detection.

## Introduction

Conotruncal heart defects (CTDs) are a diverse subtype of congenital heart diseases (CHDs) that account for approximately 25–30% of all non-syndromic CHDs. Although fetuses with CTDs typically require catheter-based or surgical treatment in the early postnatal period, the mortality rate remains high. (Bouma and Mulder, 2017). The diagnosis of CTDs at an early stage is critical for postnatal outcomes. (Holland et al., 2015; Eckersley et al., 2016; Li et al., 2016). The detection rate of prenatal CTDs has increased as screening technology has improved. There is still a lack of accurate biomarkers that can be used in addition to echocardiography to improve the detection of CTDs.

Currently, there are known factors involved in the pathogenesis of CTDs, including the genetic factors associated with cardiac development genes such as *GATA4*, *GATA6*, *TBX18*, *TBX20*, *NKX2-5*, and *SAMDS*, as well as unique copy mutations and chromosomal aneuploidy such as trisomy 13, 18, or 21, which account for 20–30% of CHDs. (Pierpont et al., 2018).

The causes of CTDs are complex; current research indicates that environmental, genetic, and epigenetic factors all contribute to the development of malformations. (Blue et al., 2012). The pathogenesis is unknown in the majority of CTDs. Methylation induces a change in the three-dimensional structure of DNA and has been shown to suppress gene transcription in the past. DNA methylation modifications may be critical in the development of CTDs such as in Tetralogy of Fallot (TOF). (Marín-García, 2009; Csáky-Szunyogh et al., 2013; and Serra-Juhé et al., 2015).

The placenta and the heart are the two organs that develop most rapidly during pregnancy. According to their developmental connections, a phenomenon called the “placenta-heart axis” has been proposed. (Maslen, 2018). Cardiovascular malformations are associated with poor trophoblast invasion and reduced transfer of nutrients to the fetus as a result of placental abnormality. (Linask, 2013). Recently, the “placenta-heart axis” has gained a considerable research attention. Moreover, studies (Radhakrishna et al., 2019 and Bahado Singh et al., 2020) have shown that the placental DNA in CHD contains a specific methylation region that is closely related to the type and occurrence of CHD and may serve as a biomarker for its detection. The purpose of this study is to characterize the abnormal changes in placental DNA methylation associated with CTDs and to assess the utility of placental DNA methylation as an epigenetic biomarker for the prediction of CTDs.

## Materials and Methods

Between September 2020 and June 2021, this prospective study enrolled 40 fetuses diagnosed with CTDs at Beijing Anzhen Hospital. Twelve fetuses were excluded due to chromosomal abnormalities. The medical Ethics Committee approved this study (note: GZR-2-006). Fetuses were classified according to the subtypes of CTDs which included TOF, double outlet right ventricle (DORV), transposition of the great artery (TGA), and pulmonary atresia with ventricular septum defect (PAVSD). All pregnant women consented to participate and signed an informed consent form. After inducing labor in fetuses, placental tissue was obtained. To ensure consistency in sampling, the placental tissue with the umbilical cord insertion part was chosen. Until DNA purification, the separated tissue blocks were stored in a refrigerator at minus 80°C. To avoid the effects of genetic malformations, Gene whole exon sequencing (GWES) analysis and copy number variation (CNV) detection were applied in all fetuses. We excluded fetuses with known or suspected genetic syndromes. As controls, 12 labor-induced fetuses with extracardiac anatomical abnormalities but no syndromic defects were used. Other obstetric complications were not noted.

QIAamp Mini Kit was used for the purification of DNA from placental tissues according to the manufacturer’s protocol. Genome-wide DNA methylation was determined using the Illumina Infinium Human Methylation 850K BeadChip (Illumina Inc., California, United States), which provides genome-wide coverage with >850000 CpG methylation sites per sample according to the manufacturer’s instructions. It is capable of producing high-quality data for DNA extracted from the samples. The genomic DNA from both the cases and the controls was subjected to bisulfite-conversion according to the manufacturers protocol using the Zymo EZ DNA Methylation kit (Zymo Research, Irvine, CA, United States). The fluorescence signals from the BeadArrays were measured using an Illumina ‘iScan’ and then analyzed using the Genome Studio software (Illumina, Inc.). The software assigned β-values to CpG probes based on the ratio of the methylated alleles (C) fluorescence signal to the sum of the methylated (C) and unmethylated (T) alleles’ fluorescence signals. Before performing detailed bioinformatics and statistical analysis, data preprocessing and quality control were performed, including examination of the background signal intensity in the affected cases and negative controls, the methylated and unmethylated signals, and the ratio of methylated and unmethylated signal intensities. The processing was carried out entirely according to the manufacturer’s protocol, and 99% of the CpG loci were unequivocally determined. The methylation status of all probes was denoted by the β-value, which is the ratio of the methylated probe intensity to the total probe intensity (sum of methylated and unmethylated probe intensities plus constant *α*, where α = 100). Between the two groups, the average β-values of the promoters were compared.

Locus quality control requires that detection p < 0.05 and Beadcount are not less than three in more than 95% of individuals. Meanwhile, loci located on X, Y chromosomes, and SNP should be removed. Individual quality control requires detection p < 0.05 in more than 95% loci. Based on the above quality control filtered data, BMIQ (Beta-mixture quantile normalization) was used to correct probe type bias to obtain the methylation level (β value) that could be used for different analyses.

### Statistical Analysis

We determined differential methylation by comparing the β-value of individual CpG loci in two groups. Based on the p-value (<0.05), the most discriminating CpG sites were identified. The R-package Limma was used to analyze the differential methylation sites, and the moderated T-statistics and empirical Bayes methods were used to test the significance of the differential sites in Limma. Bumphunters method was used to find differential methylation regions. The method first clustered according to site distance information, and then selected continuous candidate regions with consistent methylation levels in each cluster. Bumphunter constructed a linear statistical model according to candidate regions and filtered the regions with significant differences (p < 0.05). The methylation level, or β-value, of each CpG locus (one per gene), was used to calculate its predictive accuracy for CTDs and its subtype’s detection on an individual basis. The area under the receiver-operating characteristics curve was used to determine the predictive accuracy (AUC). Continuous variables are expressed as the mean ± standard deviation values. Normally distributed data were analyzed by using student’s t-test. A p value < 0.05 was considered statistically significant. The clinical data were statistically analyzed using the SPSS 22.0 software (IBM Corporation, Armonk, NY, United States).

### Gene Ontology Analysis and Functional Enrichment

KEGG Orthology Based Annotation System (KOBAS) software was used to analyze pathways, diseases, and functions at the same time. The whole analysis process consists of two steps. The first step is to map the input gene sets to the database genes and then annotate the pathways, diseases, and functions in which these gene sets are involved. In the second step, the results obtained in the previous step are compared with the background (usual genes in the whole genome, or all probes on the chip), and the statistically significant enriched pathways, diseases, or functions are mined. Statistical tests can be performed using the binomial test, chi-square test, Fisher’s exact test, or hypergeometric distribution test. Multi-hypothesis testing methods were used to adjust the p value. Genes associated with DMSs and DMRs were enriched in the Gene Ontology. Additionally, we identified overrepresented canonical pathways, biological processes, and molecular processes. We performed literature research to determine the known or plausible roles of differentially methylated genes in cardiac development.

## Results

Table 1 summarized the demographic and clinical characteristics of the cases and the controls and revealed no statistically significant differences. None of the participants experienced additional obstetric complications.

**TABLE 1:** Demographic and clinical data of cases and controls.

Characteristics	Controls	TOF	DORV	TGA	PAVSD	*p*-value
Number	12	6	10	6	6	—
Gestion week	25.83 ± 2.54	23.90 ± 3.12	24.08 ± 1.72	23.71 ± 2.55	24.50 ± 0.84	0.055
Age	29 ± 3.16	25.67 ± 4.97	27.30 ± 5.29	27.33 ± 3.56	27.17 ± 2.79	0.534

TOF: tetralogy of Fallot; DORV: double outlet right ventricle; TGA: transposition of great artery; PAVSD: pulmonary atresia with ventricular septal defect.

### Differential Methylation Sites in CTDs With Controls

**FIGURE 1:**
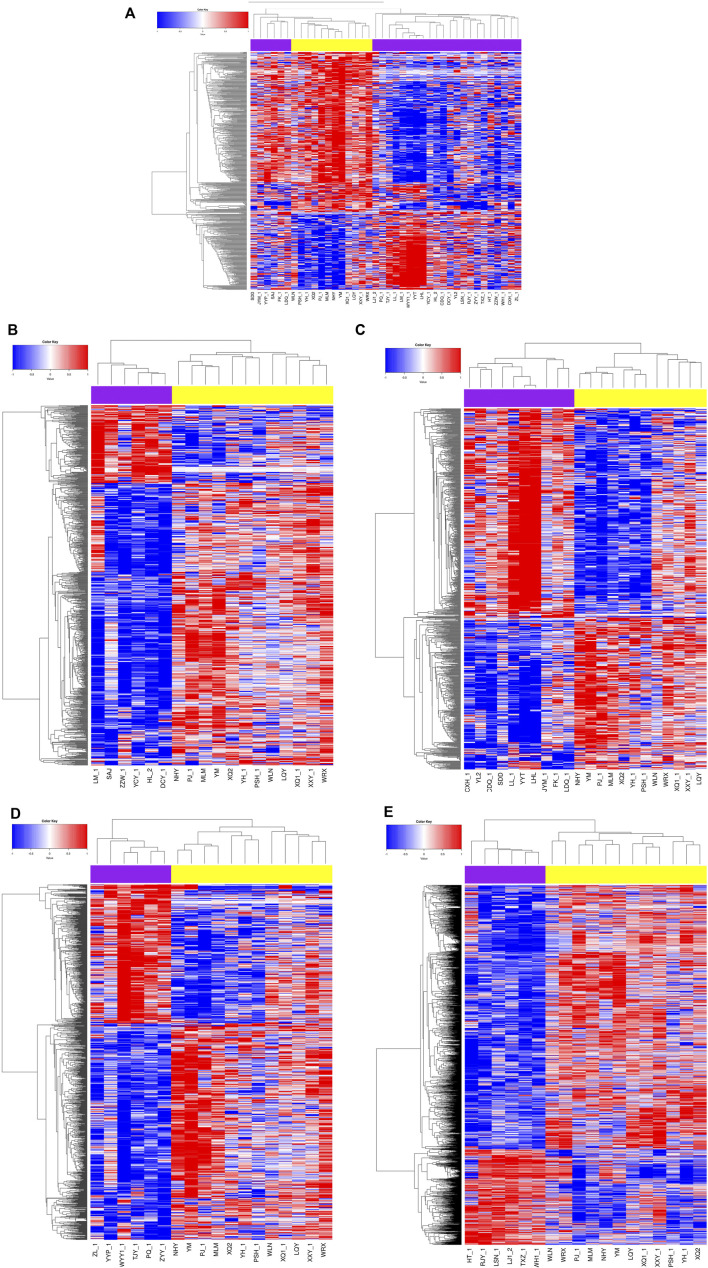
Heat map showing the differential methylation levels of CpG loci in placental DNA of cases compared with normal controls. Red represents hypermethylation, blue represents hypomethylation, the purple are the cases, the yellow are controls. **(A)** CTDs vs. controls; **(B)** TOF vs. controls; **(C)** DORV vs. controls; **(D)** TGA vs. controls; **(E)** PAVSD vs. controls.

**FIGURE 2:**
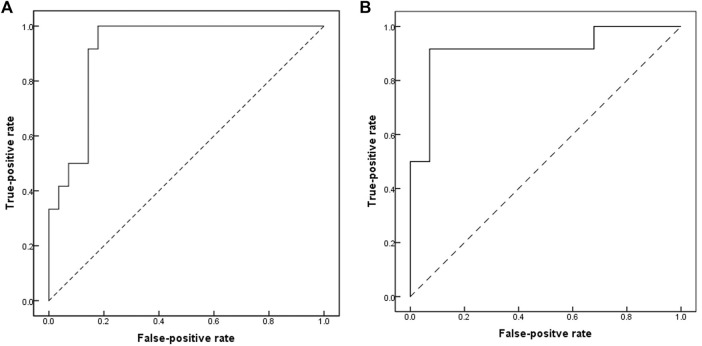
Receiver-operating-characteristics (ROC) curves showing accuracy of methylation profile of placental DNA CpG in prediction of CTDs. **(A)** cg04328562(CNN1); area under ROC curves (AUC) = 0.917 (95%CI, 0.831–1.0). **(B)** cg05167251 (chr2; HOXD9); area under ROC curves (AUC) = 0.917 (95%CI, 0.8–1.0).

In comparison to the controls, we identified 670 DMSs corresponding to 401 genes in CTDs. 444 (66.3%) of all DMSs were hypomethylated, while 226 (33.7%) were hypermethylated. The heat map in Figure 1 illustrates the differentially methylated sites in CTDs and controls, as well as subtypes of CTDs. The AUC (95%CI) and β-value for each group’s prediction were calculated. Figure 2 depicts the two genes with the highest predictive accuracy for CTDs detection (AUC >0.90). Table 2 details the most variable methylation sites between groups and the highest predictive accuracy for CTDs and their subtypes detection.

**TABLE 2:** The top DMSs between groups and AUC of epigenetic markers of each group.

	DMSs	Gene	UCSC_RefGene_Group	UCSC_CpG_Islands_Name	Relation_to_UCSC_CpG_Island	AUC (95%CI)	*p*-Value
CTDs vs. controls	cg05167251	*HOXD9*	5′UTR; 1stExon	Chr2	Island	0.914	7.11E-07
	cg04739647	*HOXD9*	5′UTR; 1stExon	Chr2	Island	0.893	5.20E-06
	cg27442308	*SOX21*	TSS1500	Chr13	Island	0.863	8.17E-06
	cg03209103	*SGCE*	Body; Body; TSS1500; Body; TSS1500	Chr7	N_Shore	0.845	1.75E-05
	cg05977002	*FLJ42709*	Body; Body	Chr5	Island	0.854	1.91E-05
	cg14991487	*HOXD9*	TSS200	Chr2	Island	0.905	1.91E-05
	c04328562	*CNN1*	1stExon; 5′UTR	Chr10	S_Shore	0.917	2.90E-05
	cg00922785	*PITX3*	TSS1500	Chr10	Island	0.881	4.23E-05
	cg18592174	*SLC18A3*	TSS1500; 5′UTR	Chr2	Island	0.899	4.45E-05
	cg01045132	*CNN1*	TSS200	—	—	0.878	5.36E-05
	cg13030331	*ANKMY1*	TSS200; TSS200	—	—	0.878	6.12E-05
TOF vs. controls	cg27624327	*SLC30A3*	Body	Chr2	Island	0.806	4.51E-07
	cg08132302	*SYT5*	3′UTR	Chr19	N_Shore	0.861	2.78E-05
	cg24951286	*PCDHB15*	TSS200	Chr5	N_Shore	1.00	4.31E-05
	cg14232289	*SSTR2*	5′UTR	Chr17	—	0.944	7.85E-05
	cg23830245	*PRR5L*	Body	Chr7	Island	0.958	0.000111
	cg00043564	*EMID2*	TSS1500	—	N_Shore	0.819	0.000112
DORV vs. controls	cg15235707	*NTM*	Body	Chr18	Island	0.967	5.84E-06
	cg17973164	*BRUNOL4 PRDM13 FAM184B ANKMY1 TMEM132D NPY2R*	TSS1500	Chr6	Island	0.958	4.33E-05
	cg17302155		Body	Chr4	Island	0.917	5.86E-05
	cg04225825		1stExon	Chr2	Island	0.950	7.30E-05
	cg13030331		TSS200	Chr12	Island	0.917	0.000118
	cg11160362		1stExon	Chr3	Island	0.933	0.00013
	cg26384430		TSS200	—	—	0.892	0.000131
TGA vs. controls	cg06587155	*HOXA11AS*	Body	Chr7	Island	0.931	4.69E-06
	cg08409173	*CLEC4GP1*	Body	Chr19	Island	0.806	6.49E-06
	cg11916729	*SCGB3A1*	TSS200	Chr5	Island	0.986	1.23E-05
	cg13876222	*NOTCH1*	Body	Chr9	Island	0.958	1.69E-05
	cg05719164	*LHX4*	Body	Chr1	Island	0.917	1.85E-05
	cg14851485	*HHIPL1*	Body	Chr14	Island	0.917	2.15E-05
	cg10042529	*CALM1*	Body	Chr1	S_Shore	1.00	2.99E-05
	cg14791413	*GLT1D1*	Body	Chr12	S_Shore	1.00	3.65E-05
	cg02192673	*NPFFR2*	1stExon; 5′UTR	Chr4	Island	0.917	3.72E-05
	cg24788999	*DCDC2C*	Body	—	—	0.958	3.88E-05
	cg00800229	*SALL2*	1stExon	—	—	0.986	4.34E-05
PAVSD vs. controls	cg10827434	*CBLB*	TSS200	Chr3	Island	0.931	9.78E-09
	cg06176124	*ZBTB45*	Body	Chr19	Island	0.847	2.56E-08
	cg20701646	*PAX6*	TSS200; 5′UTR	Chr11	Island	0.944	1.22E-07
	cg02143429	*ICE2*	TSS1500	Chr7	Island	1.00	1.89E-07
	cg07922204	*EFCAB5*	Body	Chr16		0.875	2.69E-07
	cg11399053	*SNX19*	Body			0.944	3.71E-07
	cg04135907	*LOC642943*	Body			0.944	3.94E-07
	cg21902160	*LOC646762*	Body			0.903	4.58E-07
	cg09499248	*POLR3K/SNRNP25*	Body			0.958	6.11E-07
	cg10182306	*CLCNKA*	1stExon; TSS1500 body			0.931	6.71E-07

DMSs: differential methylated sites; TOF: tetralogy of Fallot; DORV: double outlet right ventricle; TGA: transposition of a great artery; PAVSD: pulmonary atresia with a ventricular septal defect.

Furthermore, when comparing CTDs and controls, we identified 29 DMRs. Three DMRs were hypermethylated and 26 were hypomethylated. The majority of the DMRs were located on the CpG island. In comparison to the controls, we identified 20, 6, 27, and 17 DMRs in TOF, DORV, TGA, and PAVSD, respectively. We discovered that the majority of DMRs in these cases were hypermethylated. Table 3 lists the gene-related sites and their locations. *NR2E1* and *ECE1* were identified in more than two groups of gene-related sites.

**TABLE 3:** Genes with differential methylation regions in each group.

—	CTDs	TOF	DORV	TGA	PAVSD
Number of DMRs (hypermethylated, %)	29 (78.3%)	20 (20, 100%)	6 (67%)	27 (59.4%)	17 (94.1%)
TSS1500	*NPR3*	*KCNMA1*	—	*HSPA1A*	*S1PR1*
	*NR2E1*	*TRIM26*		*SALL1*	*NR2E1*
	*SLC18b1*	*HOXA5*		*NR2E1*	*GFRA1*
	*C6orf192*			*PDK4*	*SALL1*
	*CLIP4*			*HOXA7*	*SNCA*
	*RNF135*			*GDNF*	
	*ZSCAN12*			*MEGF10*	
				*FOXG1*	
TSS 200	*CDKN1C*	*C11orf97*	*C15orf56*	*SLC37A2*	*ADORA2B*
	*ADORA2B*			*MYOCD*	
	*DGKZ*			*LEKR1*	
	*RHOBTB1*			*ALDH1A2*	
	*MIR886*	*SEC31B*		*CYP2R1*	
	*GPR150*			*NELL1*	
	*EPSTI1*	*NELL1*		*SAR1B*	*ALDH1A2*
	*THAP3*			*SMOC1*	
	*LOX*	*TMEM232*	*RIMS1*	*MIR196B*	
	*C13orf38*				*PCK2*
	*LOC100302652*	*WSCD2*			
	*GPR75*				
5′UTR	*LM O 3*	*S100A13*	*PAK6*	*ADRB2*	*C6orf47*
	*FAM176A*	*ART5*		*TTC23*	*TTC23*
	*GUCY1A3*				*PAK6*
					*FAM198A*
EXON1	*LOX*	*CA13*	*P4HTM*	*FAM198A*	*KIAA1826*
				*TLL1*	*LOX*
GENE BODY	*HLA-J; NCRNA00171*	*ECE1*	*ECE1*	*C12orf10*	*TLL1*
	*HCG4*	*CRABP1*		*ECE1*	
		*C12orf10*		*TMED7-TICAM2*	
		*ZNF252*		*PENK*	
		*C8orf77*		*CRABP1*	
		*PRDM1*			
		*ARID5B*			
		*HLA-DPB1*			
ISLANG	chr12:22093954–22095357	chr11:94245546–94245891	—	—	chr10:118608599–118609377
NSHORE	chr5:135416204–135416475	—	chr12:29302034–29302954	chr12:29302034–29302954	—

DMRs: differential methylated regions; CTDs: conotruncal heart defects; TOF, tetralogy of Fallot, DORV: double outlet right ventricle; TGA: transposition of giant arteries; PAVSD: pulmonary atresia with ventricular septum defect.

### Gene Ontology and Pathway Enrichment Analysis of CTDs With Controls

**FIGURE 3:**
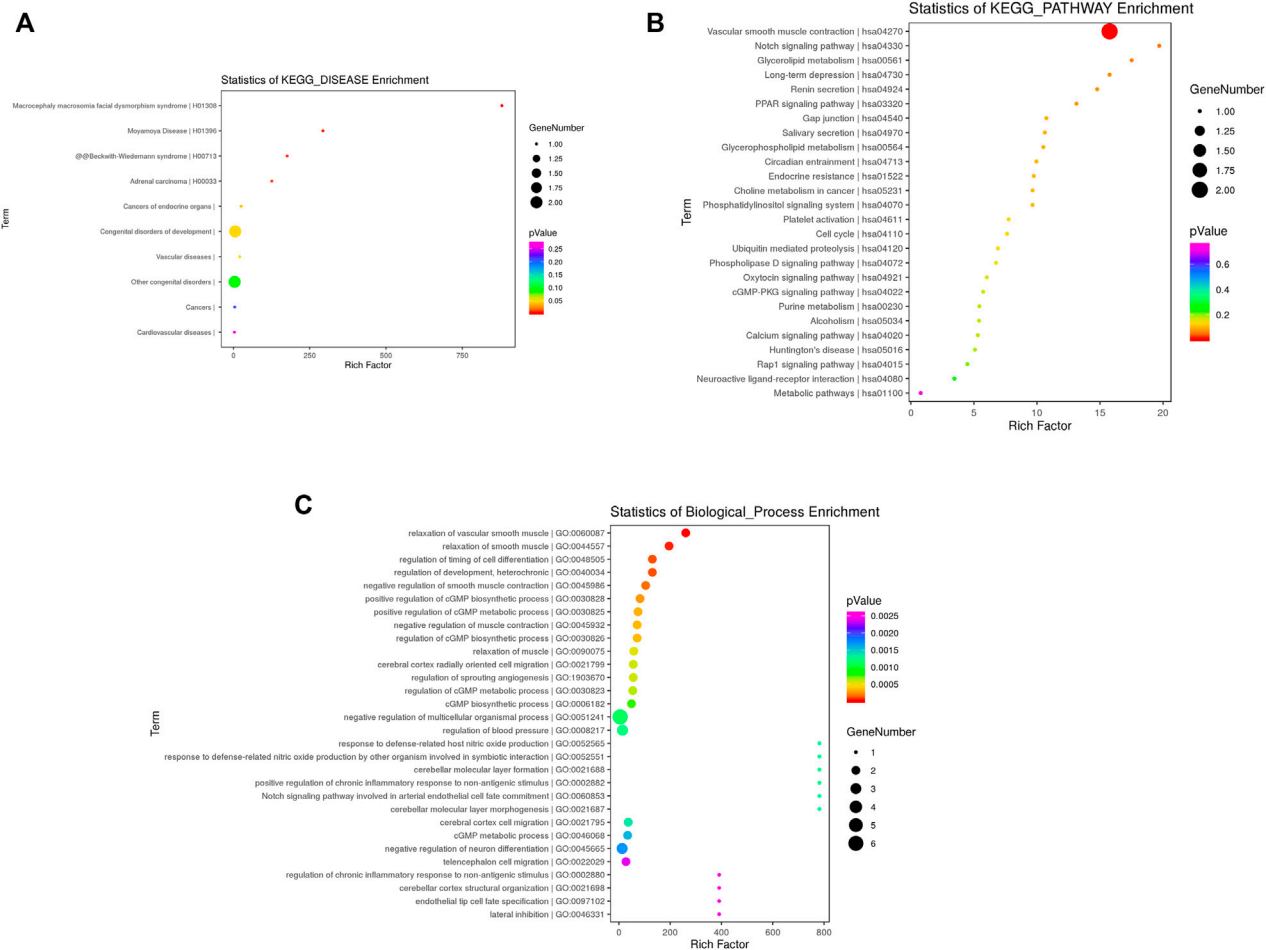
Functional enrichment analysis of differential methylation regions annotated genes in CTDs vs. controls KEGG enrichment analysis in disease **(A)** and pathway **(B)**; **(C)** Top 30 terms of biological process (BP).

Between CTDs and controls, disease ontology analysis, go enrichment analysis, and pathway analysis of the DMRs were performed. Figure 3 summarizes the top 30 items. Following our cases, disease ontology revealed that the DMRs of CTDs were associated with congenital developmental disorders, vascular diseases, and cardiovascular diseases. CTDs were associated with a variety of pathways, including vascular smooth muscle contraction, Notch signaling, glycerolipid metabolism, long-term depression, renin secretion, *PPAR* signaling, gap junctions, salivary secretion, circadian entrainment, and endocrine resistance. *CNN1* was found to be involved in smooth muscle contraction; *NOTCH1* was found to be involved in the *NOTCH* signaling pathway.

## Discussion

Even with today’s highly developed diagnostic methods, early detection and classification of CHD remain a significant clinical challenge. Epigenetic biomarkers of CHD are of great clinical interest and the predictive value of DNA methylation changes in CHD has been demonstrated in numerous studies. Since the placenta and the heart develop in the same way, the placenta may be an ideal tissue for both understanding the mechanism of CHD and developing biomarkers for it. Several studies (Fantasia et al., 2018; Fantasia et al., 2019; and Binder et al., 2020) have demonstrated placental dysfunction in the absence of impaired placental perfusion in pregnancies with isolated CHD, reinforcing the preceding position. Additionally, the placenta is available during the first to third trimesters of pregnancy.

We identified several potential methylation markers for the prediction of CTDs in our study. The best predictive performance (AUC >0.90) was achieved by *HOXD9* and *CNN1*. To gain a better understanding of the function of *HOXD9* and *CNN1*, we investigated the human protein atlas (Uhlén et al., 2015) database. *HOXD9* is not restricted to the placenta or the heart but is expressed in multicellular organisms, with a high level of expression in the endometrium. According to the latest published data, it plays a critical role in the promotion of carcinoma (Zhu et al., 2019 and Wen et al., 2020) and arterial inflammation (Souilhol et al., 2020). However, the role of *HOXD9* in the development of the heart and placenta remains unknown. *CNN1* is expressed in the cytoplasm of smooth muscle and myoepithelial cells with a high degree of specificity. Additionally, *CNN1* encodes a protein that plays a role in the regulation and modulation of smooth muscle contraction. It also plays a role in the development of cardiomyopathy (Lu et al., 2014), placental vascular development, and tissue morphology (de Barros Mucci et al., 20052020). Further studies will be critical to elucidate its role in the pathogenesis of CHD.

Additionally, we identified methylation predictors in subtypes of CTDs, including TOF, DORV, TGA, and PAVSD, as shown in Table 2. The intriguing thing is that the genes with the highest prediction for each group are distinct, although these cases are classified as CHD in anatomy. The most frequently studied difference between these cases is the methylation changes in TOF. Bahado-Singh. R *et al.* reported that methylation changes in *ARHGAP22*, *CDK5*, *TRIM27*, and *IER3* in the placenta are excellent individual predictive markers of TOF, a finding that was not replicated in our study. Different gestation weeks and tissue preservation in the cases may be the primary reasons. *PCDHB15*, *SSTR2*, and *PRR5L* were identified in TOF in our study. *PCDHB15* has a low tissue specificity and its specific functions are unknown, but it is highly likely to involve in the establishment and function of specific cellcell neural connections (Lee and Lee, 2008). *SSTR2* expression in the heart from day 14–15 with a peak at day 17 was discovered in a study (Ludvigsen et al., 2015) of mouse embryos. This coincides with the development of the heart and the placenta. It is not, however, specified in these two organs. There is insufficient evidence to suggest that it plays a role in the development of cardiovascular disease. The *PPR5L* protein is associated with the *mTORC2* complex, which regulates cellular processes such as survival and organization of the cytoskeleton. (Pearce et al., 2007). It acts as a substrate-specific regulator of the *mTORC2* complex, preventing, for example, the specific phosphorylation of PKCs and eventually controlling the cell migration. (Gan et al., 2012)*mTORC2* is required for cardiomyocyte differentiation from embryonic stem cells (Zheng et al., 2017), postnatal heart growth, and heart function maintenance in mice (Zhao et al., 2014). Nevertheless, the molecular mechanism by which *PPR5L* functions in CHD is unknown and requires further investigation. *NTM*, *BROUNL4*, *PRDM13*, *FAM184B*, *ANKMY1*, and *TMEM132D* were identified as DMSs genes in the DORV group. Both of them were not tissue-specified and had unknown functions during heart development. Among these TGA group DMSs, *NOTCH1*, and *NPFFR2* have been associated with heart development. Some studies (High et al., 2009 and Koenig et al., 2016) established the role of endothelial *NOTCH1* in the proper development of semilunar valves, cardiac outflow tract remodeling, and aortic arch artery remodeling. Transposition of the great arteries is associated with abnormal outflow tract development. A study (Kerstjens-Frederikse et al., 2016) found that cardiovascular malformation caused by *NOTCH1* mutation does not keep left. The phenotypic spectrum included CTDs, which was consistent with our findings. *NOTCH1* was recently identified as a major candidate gene for TOF in a whole-exome sequencing study conducted by (Page et al., 2019) While the highest concentration of *NPFF* receptor 2 (*NPFFR2*) mRNA is found in the placenta, a recent study (Zhu et al., 2020) demonstrated that *NPFF* acts via *NPFFR2* to increase HCG-β production and promotes GCM1-dependent expression of syncytin one and two in cytotrophoblasts. Trophoblast cells are critical in the formation of placental blood vessels. In the PAVSD group, several genes were identified as the predictive makers, but none of them were specified as expressed in the placenta or heart in the human protein atlas. As discussed previously, methylation changes in placental tissue may serve as predictive biomarkers for CHD, even when differentiating between different subtypes.

In comparison to the controls, our study identified 29, 20, 6, 27, and 17 DMRs in CTDs, TOF, DORV, TGA, and PAVSD, respectively. The majority of them had been hypermethylated. Among the genes with differential methylation regions (Table 3), *NR2E1* located at transpositional start site (TSS) 1500 and *ECE1* located within the gene body were found in more than two groups. *NR2E1* mRNA has been detected in a variety of organs, including the placenta, but its precise function is unknown. Endothelin-converting enzyme-1 (*ECE1*) is required for the development of a subset of neural crest lineages, including cardiogenesis, and a case-control study (Wang et al., 2012) suggested that *ECE1* polymorphisms may contribute to susceptibility to sporadic CHD in the Chinese population, particularly in TOF and peri membranous ventricular septal defect (pmVSD). In our study, we also discovered differential methylation of *ECE1* in TOF. As a result, additional research is required to elucidate the mechanisms. Consequently, the subtypes of CTDs may share some common differential methylation genes. However, distinct subgroups may have distinct differential methylation genes that influenced the development of the heart. Due to the interaction of all of these genes, distinct subtypes of CTDs exist.

Our study had some limitations. To begin, only a few cases were included, especially in the subtype group. Additional research is required in a large number of cases. Second, one should consider the effect of gestational age on methylation. Additionally, we did not examine the levels of gene expression (mRNA or protein), which is the ultimate determinant of the biological consequence of DNA methylation. Moreover, our specimens were all derived from fetuses undergoing labor induction, so we could not observe their efficacy in predicting pregnancy outcome or even post-birth prognosis. These are the things we need to improve next.

In conclusion, our study established that abnormal changes in placental methylation are associated with CTDs and may be predictive of CTDs. Numerous altered genes are already known or suspected to play a role in cardiovascular or placental development, additional research is planned to corroborate our findings.

## Data Availability

The original contributions presented in the study are included in the article/Supplementary Material, further inquiries can be directed to the corresponding authors.
